# Community acquired *Acinetobacter baumannii* in pediatric patients under 1 year old with a clinical diagnosis of whooping cough in Lima, Peru

**DOI:** 10.1186/s13104-021-05826-y

**Published:** 2021-11-10

**Authors:** Isaac Peña-Tuesta, Cristina del Valle-Vargas, Veronica Petrozzi-Helasvuo, Miguel Angel Aguilar-Luis, Hugo Carrillo-Ng, Wilmer Silva-Caso, Juana del Valle-Mendoza

**Affiliations:** 1 School of Medicine, Research Center of the Faculty of Health Sciences, Universidad Peruana de Ciencias Aplicadas, Lima, Peru; 2 Laboratorio de Biologia Molecular, Instituto de Investigación Nutricional, Lima, Peru; 3 Department of Medicine, School of Medicine, Universidad de Barcelona, Barcelona, Spain

**Keywords:** *A. baumannii*, Acute respiratory infections, ARI, *Bordetella pertussis*, Peru

## Abstract

**Objective:**

This study aimed to determine the prevalence of *A. baumannii* in children aged less than 1 year admitted with a clinical diagnosis of whooping cough.

**Results:**

A total of 225 nasopharyngeal samples from children under 1 year old hospitalized with clinical diagnosis of whooping cough were studied from January 2010 to July 2012. The presence of *A. baumannii* was detected in 20.89% (47/225) of the nasopharyngeal swab samples. Among the 47 patients with *A. baumannii*: 5 were diagnosed with *A. baumannii* monoinfection, 17 co-infection with bacteria, 7 co-infection with virus and 18 co-infection with bacteria + virus. It was observed that 51.6% (116/225) were children between 29 days and 3 months old, this same group had the highest overall prevalence with 53.3%. The most common co-infecting pathogens were *Bordetella pertussis* in 55.3%, *Adenovirus* in 42.6% and *Mycoplasma pneumoniae* in 23.4%.

**Supplementary Information:**

The online version contains supplementary material available at 10.1186/s13104-021-05826-y.

## Introduction

*Acinetobacter* is an opportunistic gram-negative coccobacilli bacterial family associated with a wide variety of hospital-acquired infections, often transmitted to patients via persistence on environmental surfaces and transient colonization of the hands of health care workers [1–3]. *Acinetobacter baumannii* is the most virulent of all *Acinetobacter* species, being nosocomial pneumonia its most common and lethal clinical presentation [4, 5]. The widespread resistance of these bacteria against a variety of antibiotics has become a major threat for public health [1]. The main predisposing factors for infection are colonization pressure, selection by exposure to broad-spectrum antibiotics and disruption of anatomical barriers. Consequently, immunocompromised patients are the most vulnerable, with higher incidence of infection occurring in intensive care units (UCIs), long-term hospitalized patients or patients undergoing major invasive procedures [5]

In Peru, acute respiratory infections (ARIs) continue to be the leading cause of premature mortality. For example, in the year 2019 a total of 2,501,436 cases of acute respiratory infections were registered in children under 5 years old, being the ones younger than 1 year old the most commonly affected [6]. A great variety of pathogens have been detected in children with ARI in Peru. For example a previous study reported that the most common viruses in children with ARI were RSV-A, followed by influenza A, Parainfluenza-1 and 2 [7]. Also, other studies have reported a high prevalence of atypical bacteria such as *Mycoplasma Pneumonia*, *Chlamydia pneumoniae* and *Bordetella pertussis* in pediatric patients with ARI [8, 9]. Among acute respiratory infections, whooping cough remains a large cause of morbidity and mortality in Peru. In a previous study [10], *B. pertussis* was detected in 39.54% children with whooping cough, being more frequent in those younger than 3 months old. However, many children did not have a etiological diagnosis and other respiratory pathogens may be involved*.* Data on the epidemiology of *A. baumannii* and its role in respiratory infections among pediatric patients in Peru is still scarce. Therefore, the aim of this study is to determine the prevalence of *A. baumannii* in children aged less than 1 year old hospitalized for whooping cough.

## Main text

### Materials and methods

#### Patients and study design

The current study was conducted as part of a previous study evaluating the epidemiology of respiratory pathogens in children younger than 1 year old hospitalized with whooping cough [10]. In brief, a prospective cross-sectional study was conducted in five hospitals in Lima, Peru from January 2010 to July 2012. The study regions had a representative population, since Lima and Cajamarca have high rates ARIs cases.

#### Samples

225 samples of children younger than 1 year old were included for analysis. For this analysis, the remnants of the respiratory samples obtained from a previous study [10]. We could only analyze 225/392 samples due to insufficient or inadequate state of the conservation of the remaining isolates.

#### Ethics statement

This study was approved by the Research Ethics Board of the Hospital Docente Regional de Cajamarca, Peru. Samples were collected after a parent or guardian signed the informed consent to agree to participate, the informed consent was taken as a part of a previous study [10], which allowed the use of the samples for future studies. All procedures were performed under the international ethics guidelines for research in human healthcare by the Council for International Organizations of Medical Sciences (CIOMS) and the World Health Organization (WHO).

#### DNA extraction

DNA was extracted from a volume of 200 µL of each sample using a commercial kit (*High Pure Template Preparation Kit; Roche Applied Science*, Germany) according to the manufacturer's instructions.

#### PCR amplification

The presence of *A. baumannii* was determined using a real-time PCR assay for the gen OXA-51, an independent region of the *A. baumannii* genome describe by Chen et al. [11] (Additional file 1: Table S1).

The product *LightCycler FastStart DNA Master HybProbe* (*Roche Applied Science*, Germany) was used to prepare the reaction mixture which contained 10.6 µL of free DNAsa/RNAsa’s water, 2.4 µL of Mg^+2^ solution in a concentration of 25 mM, 2.0 µL of the Faststart enzyme, 1 µL of each specific primer for OXA-51 in a 1 mM concentration, 1 µL of the specific quencher and 2 µL DNA. The amplifications conditions were 95 °C for 10 min followed by 55 cycles of 95 °C for 5 s, 60 °C for 5 s and 72 °C for 15 s. All procedures were performed in a LightCycler 2.0 instrument and data were analyzed with LightCycler software 4.1 (Roche Diagnostics, Mannheim, Germany).

#### Statistical analysis

Qualitative variables were reported as frequencies and percentages. All analyses and graphs were carried out using the GraphPad 9.1.0 software (San Diego, CA, USA).

### Results

A total of 225 nasopharyngeal samples from children under 1 year old hospitalized with clinical diagnosis of whooping cough were studied from January 2010 to July 2012. The presence of *A. baumannii* was detected in 20.89% (47/225) of the nasopharyngeal swab samples by the Real Time PCR of the OXA-51 gene. Additional file 2: Fig. S1 shows the distribution of infection pattern among the patients with *A. baumannii* diagnosis*.* Additional file 2: Fig. S1A shows that among the 47 patients with *A. baumannii*: 5 were diagnosed with *A. baumannii* monoinfection, 17 co-infection with bacteria, 7 co-infection with virus and 18 co-infection with bacteria + virus. In the case of co-infections patients were diagnosed with up to six pathogens at the same time, as shown in Additional file 2: Fig. S1B.

Table 1 shows the demographic characteristics of our study population. It was observed that 51.6% (116/225) were children between 29 days and 3 months old, this same group had the highest overall prevalence with 53.3%. No significant difference in gender was observed in the distribution of our studied population nor in the prevalence of positive cases of *A. baumannii.* Moreover, the evaluation of household contacts was also performed. Most of the patients had contact with their respective mother (20.4%) and siblings younger than 10 years old (24.4%). In the case of patients positive for *A. baumannii* the proportion of patients that had contact with an ill mother was (29.8%) compared to patients without *A. baumannii* (17.9%).

**Table 1:** Prevalence and demographic characteristics of the the patients studied

Characteristics	Total patients		Patients positive for *A. baumannii*		Patients negative for *A. baumannii*	
	Frequency n = 225 (%)	Prevalence (%)	Frequency (n = 47)	Prevalence (%)	Frequency (n = 178)	Prevalence (%)
Age						
≤ 28 days	14	6.2	3	6.4	12	6.7
29 days–< 3 months	116	51.6	25	53.3	91	51.1
3– < 6 months	63	28.00	12	25.4	50	28.1
6–12 months	32	14.2	7	14.9	25	14.1
Sex distribution						
Male	119	52.8	25	53.2	94	52.8
Female	106	47.2	22	46.8	84	47.2
Patient setting						
Inpatient	202	89.78	43	91.49	159	89.33
ICU	23	10.22	4	8.51	19	10.67
Nutritional status						
Malnutrition	13	5.8	2	4.3	11	6.2
Obesity	3	1.3	1	2.1	2	1.1
Household contacts						
Mother	46	20.4	14	29.8	32	17.9
Father	16	7.1	4	8.5	12	6.7
Siblings ≤ 10 years old	55	24.4	7	14.9	48	26.9
Siblings ≥ 10 years old	6	2.7	2	4.3	4	2.2
Uncles/Aunts	26	11.6	10	21.3	16	8.9
Others	3	1.3	1	2.1	2	1.1

Table 2 shows the most frequent clinical signs and symptoms, as well as the clinical outcomes in patients according to their infection status. The clinical manifestations between the groups with infection by *A. baumannii* as the only identified microorganism and *A. baumannii* in coinfection with other bacteria or viruses do not show many differences. A low frequency of fever is observed in the *A. baumannii* + virus group. Difficulty breastfeeding and cough paroxysms occurred in all infants with *A. baumannii* monoinfection and all those coinfected with *A. baumannii* virus presented respiratory distress. Patients that were admitted to ICU were 1 in the *A. Baumannii* + bacteria group, 1 in the *A. Baumannii* + virus and 2 in the *A. Baumannii* + bacteria + virus group. Mortality occurred in 2 cases of *A. Baumannii* + bacteria + virus and in 1 case of *A. Baumannii* + bacteria.

**Table 2:** Clinical signs and clinical outcomes in PCR confirmed cases of *Acinetobacter baumannii* according to pattern of infection

	*A. Baumannii monoinfection*		*A. baumannii* + *bacteria*		*A. baumannii* + *virus*		*A. baumannii* + *virus* + *bacteria*		Negative for *A. baumannii*	
	Frequency (n = 5)	Prevalence (%)	Frequency (n = 17)	Prevalence (%)	Frequency (n = 7)	Prevalence (%)	Frequency (n = 18)	Prevalence (%)	Frequency (n = 178)	Prevalence (%)
Clinical sign										
Paroxysmal coughing	5	100.0	14	82.4	5	71.4	18	100.0	153	68.0
Respiratory distress	4	80.0	12	70.6	7	100.0	14	77.8	135	60.0
Rubicundity	4	80.0	11	64.7	5	71.4	15	83.3	126	56.0
Cyanosis	4	80.0	12	70.6	5	71.4	8	44.4	103	45.8
Post tussive emesis	3	60.0	5	29.4	3	42.9	11	61.1	84	37.3
Difficulty in breastfeeding	5	100.0	7	41.2	6	85.7	9	50	80	35.6
Fever	3	60.0	5	29.4	2	28.6	8	44.4	66	29.3
Inspiratory stridor	4	80.0	4	23.5	1	14.3	7	38.9	44	19.6
Sleep apnea	2	40.0	4	23.5	0	0.0	4	22.2	24	10.7
Dhiarrea	0	0.0	2	11.8	0	0.0	2	11.1	19	8.4
Clinical outcome										
Broncho-obstructive syndrome	4	80.0	9	52.9	6	85.7	12	66.7	98	43.6
Pneumonia	2	40.0	1	5.9	4	57.1	6	33.3	48	21.3
Atelectasis	1	20.0	1	5.9	2	28.6	2	11.1	23	10.2
Umbilical hernia	0	0.0	0	0.0	1	14.3	3	16.7	2	0.9
Seizures	0	0.0	0	0.0	0	0.0	1	5.6	2	0.9
ICU admission	0	0.0	1	5.9	1	14.3	2	11.1	0	0.0
Death	0	0.0	1	5.9	0	0.0	2	11.1	0	0.0

Finally, an evaluation of the bacterial and viral co-infections was performed, as shown in Table 3. It was observed that in only 10.6% (5/47) of the patients *A. baumannii* was the detected as a single pathogen. The groups with the higher proportion of patients was the one with two infectious agents (*A. baumannii* + bacteria*)* with 25.5% and the ones with three infectious agents (*A. baumannii* + bacteria + virus) with 27.5%. We could highlight that 26/47 (55.3%) of pediatric patients with whooping cough were diagnosed with *A. baumannii* in addition to *B. pertussis.* Followed by *Mycoplasma pneumoniae* coinfection in 11/47 cases (23.4%). In the case of viral pathogens, the most frequently co-detected was adenovirus with 20/47 (42.6%).

**Table 3:** Bacterial and viral co-detection in positive children with *A. baumannii* with a clinical diagnosis of whooping cough

_Etiologic agents_			_N_ _(47)_	_%_	_Total_ _%_
_Number_	_Type_	_Species_			
_One-infectious agent_	_Bacterial_	_*A. baumannii*_	_5_	_10.6_	_10.6_
_Two-infectious agents_	_Bacterial_	_*A. baumannii* + *M. pneumoniae*_	_4_	_8.5_	
		_*A. Baumannii*+ *B. pertussis*_	_8_	_17_	_25.5_
	_Bacterial–Viral_	_*A. baumannii* + ADV_	_2_	_4.3_	_10.6_
		_*A. baumannii* + Flu-B_	_1_	_2.1_	
		_*A. baumannii* + PIV-1_	_1_	_2.1_	
		_*A. baumannii* + PIV-3_	_1_	_2.1_	
_Three-infectious agents_	_Bacterial_	_*A. baumannii* + *C. pneumoniae* + *M. pneumoniae*_	_1_	_2.1_	_8.5_
		_*A. baumannii* + *C. pneumoniae* + *B. pertussis*_	_1_	_2.1_	
		_*A. baumannii* + *M. pneumoniae* + *B. pertussis*_	_2_	_4.3_	
	_Bacterial-Viral_	_*A. baumannii* + *C. pneumoniae* + RSV-A_	_1_	_2.1_	_27.5_
		_*A. baumannii* + *M. pneumoniae* + ADV_	_1_	_2.1_	
		_*A. baumannii* + *B. pertussis* + ADV_	_8_	_17_	
		_*A. baumannii* + *B. pertussis* + Flu-B_	_1_	_2.1_	
		_*A. baumannii* + ADV + Flu-B_	_1_	_2.1_	
		_*A. baumannii* + ADV + Flu-A_	_1_	_2.1_	
_Four-infectious agents_	_Bacterial_	_*A. baumannii* + *C. pneumoniae* + *M. pneumoniae* + *B. pertussis*_	_1_	_2.1_	_2.1_
	_Bacterial–Viral_	_*A. baumannii* + *C. pneumoniae* + ADV + RSV-A_	_1_	_2.1_	_8.5_
		_*A. baumannii* + *B. pertussis* + ADV + Flu-B_	_2_	_4.3_	
		_*A. baumannii* + *M. pneumoniae* + ADV + Flu-B_	_1_	_2.1_	
_Five-infectious agents_	_Bacterial-Viral_	_*A. baumannii* + *C. pneumoniae* + *B. pertussis* + ADV + Flu-B_	_1_	_2.1_	_4.2_
		_*A. baumannii* + *B. pertussis* + ADV + Flu-A + Flu-B_	_1_	_2.1_	
_Six-infectious agents_	_Bacterial-Viral_	_*A. baumannii* + *C. pneumoniae* + *M. pneumoniae* + *B. pertussis* + ADV + Flu-B_	_1_	_2.1_	_2.1_

### Discussion

Interest in *Acinetobacter baumannii* has been growing for the past years due to the emergence of multiresistant strains, that affect particularly adult patients with prolonged hospital stays and major procedures [12]. However, in hot and humid areas, such as tropical countries, infections by this pathogen can be community-acquired [12, 13].

Currently, the epidemiology of *A. baumannii* in pediatric patients have been a topic of great interest due to the increasing rates of infection and antibiotic resistance [14]. Infection by this pathogen predominantly affect pediatric patients, causing detrimental outcomes on their health, such as prolonged ICU stays and death [15, 16]. Previous studies show that this pathogen infects pediatric critically ill patients, with underlying diseases, immune deficiency, and invasive operations [15]. Also, outbreaks have been reported in neonatal and pediatric intensive care units, as well as burn units [16]. This pathogen has been also been associated to lower respiratory tract infection in pediatric patients with tracheostomy [17]. This pathogen causes serious disease due to the presence of several virulence factors, including porins, lipopolysaccharides, capsular proteins, among others. These have been associated with adherence and invasion properties of this bacteria, biofilm formation and evasion of the host immune response [18]. On the other hand, its ability to develop multiple antibiotic resistance mechanisms have rendered most antibiotics useless. The expression of metallo beta-lactamases, carbapenemases and efflux pumps contribute to the resistance towards the most important antibiotics in the treatment of this bacteria [19, 20].

The epidemiology of this pathogen has not been extensively studied in children with non-serious illness outside of intensive care units. Therefore, we performed the first study evaluating the presence of *Acinetobacter baumannii* in pediatric patients under 1 year old with a clinical syndrome of whooping cough. The current study was carried out as part of a previously published report [10], in which the presence of *Bordetella pertussis* was detected in 155/392 (39.54%) children under 1 year old with whooping cough; nonetheless, more than half of the patients remain undiagnosed. Of the total 392 samples collected previously, we could only analyze 225 due to insufficient or inadequate state of conservation of the remaining 167 isolates. We report a prevalence of 20.89% of *A. baumannii*, which corresponds to 47 cases of the total 225 nasopharyngeal samples used for this study. The age group with the greater number of cases were young infant between 29 days to < 3 months, followed by the 3 months–< 6 months, these findings highlight the need to maintain a high clinical suspicion particularly in this age groups. We suspect that most of the patients were colonized by community-acquired *A. baumannii,* given that samples were taken upon admission to hospitalization*.* The presence of *A. baumannii* in relation to whooping cough have also been reported previously, for example, it has been detected in children with whooping cough as a complication, causing severe pertussis and pneumonia [21]. However, this is the first report of this pathogen in young pediatric patients with community-acquired *A. baumannii.*

In the present study we found that *A. baumannii* infection may be detected in addition to other respiratory viral and bacterial pathogens, with up to six infectious agents in the same patient. We found that *A. baumannii* monoinfection was only detected in 5 patients. The most frequent co-detected pathogens were *Bordetella pertussis*, *Mycoplasma pneumoniae* and *Adenovirus*. The nasopharynx of pediatric patients can harbour a great variety of different viruses and bacterial species. Under different circumstances of health and disease, a certain microorganism may be pathogenic and others only colonizers [22, 23]. In the current study the majority of patients had co-detection of at least two infectious agents; however, not all of them necessarily contribute to the development of disease and some may be only colonizers. We could highlight the high frequency of co-detection between *B. pertussis* and *A. baumannii,* which was detected in 55.3% of the total samples analyzed. The co-infection between these pathogens has not been reported previously, also the presence of *A. baumannii* was higher than expected in nasopharyngeal samples from patients with whooping cough and may have a role in the pathogenesis of this disease.

Finally, we performed an analysis of the clinical symptoms and outcomes between the groups with different patterns of infection, to determine if co-infections contribute to distinct clinical features. Clinical symptoms were similar between the different patterns of infections; however, clinical outcomes such as broncho-obstructive syndrome, pneumonia, death and ICU admission, were more frequent in the groups of *A. baumannii* with co-infections compared to patients without *A. baumannii*. These findings may indicate that co-infections between *A. baumannii* and other respiratory pathogens may lead to worse outcomes. Similarly, a previous review of viral bacterial co-infection of the respiratory tract demonstrates that co-infections are associated with detrimental effects on the patients such as higher risk to develop more severe disease [22].

In conclusion, this is the first report describing the presence of community-acquired *A. baumannii* in nasopharyngeal samples of children under 1 year old with whooping cough. Co-detection with multiple pathogens is frequent, with up to 6 respiratory infectious agents at the same time, being *A. baumannii* and *B. pertussis* co-infection the most prevalent. Further studies are required to determine the role of *Acinetobacter baumannii* infections in acute respiratory infections in young children, particularly in children with non-critical disease and outside of intensive care units.

## Limitations

Firstly, an important limitation is that resistance to *A. baumannii* was not evaluated. Also, general and specific genotype distribution of *A. baumannii* varies mong geographic areas, therefore, it is important to carry out surveillance studies of this pathogen and clonality.

## Supplementary Information

**Additional file 1: Table S1.** Primers and probe for the detection of the OXA-51 gene of *Acinetobacter baumannii.*

**Additional file 2: Fig. S1.** A: Pattern of infection in the total population. B: Co-detection of pathogens in *Acinetobacter baumannii* positive children.

## Data Availability

Abstraction format used in the study and dataset are available and accessible from the corresponding author upon request in the link: https://figshare.com/s/6990881fe600d0a27881

## Abbreviations

ARIs: Acute respiratory infections
UCIs: Intensive care units
PCR: Polymerase chain reaction
DNA: Deoxyribonucleic acid
bp: Base pairs
