# Supplementary material for: Community acquired Acinetobacter baumannii in pediatric patients under 1 year old with a clinical diagnosis of whooping cough in Lima, Peru
Source: BMC Res Notes. 2021 Nov 10;14:412. doi: 10.1186/s13104-021-05826-y (PMC8579657; doi:10.1186/s13104-021-05826-y)
Supplement: Supplementary file 1 — Additional file 1: Table S1. Primers and probe for the detection of the OXA-51 gene of Acinetobacter baumannii. [file 13104_2021_5826_MOESM1_ESM.docx]

**Table S1.** Primers and Probe for detection gen OXA-51 of *Acinetobacter baumannii*.

| Name | Sequence (5’-- 3’) | Size (bp) | Ref |
| --- | --- | --- | --- |
| Forward OXA-51 | TTTAGCTCGTCGTATTGGACTTGA | 108 bp | [11] |
| Reverse OXA-51 | CGGAGAAGGACCCACCAGCCAAAA |  |  |
| Probe  OXA-51 | FAM-TGGCAATGTAGATATCGGTACCCAAGTC-TAMRA |  |  |
