# Supplementary figures and images for: Community acquired Acinetobacter baumannii in pediatric patients under 1 year old with a clinical diagnosis of whooping cough in Lima, Peru
Source: BMC Res Notes. 2021 Nov 10;14:412. doi: 10.1186/s13104-021-05826-y (PMC8579657; doi:10.1186/s13104-021-05826-y)

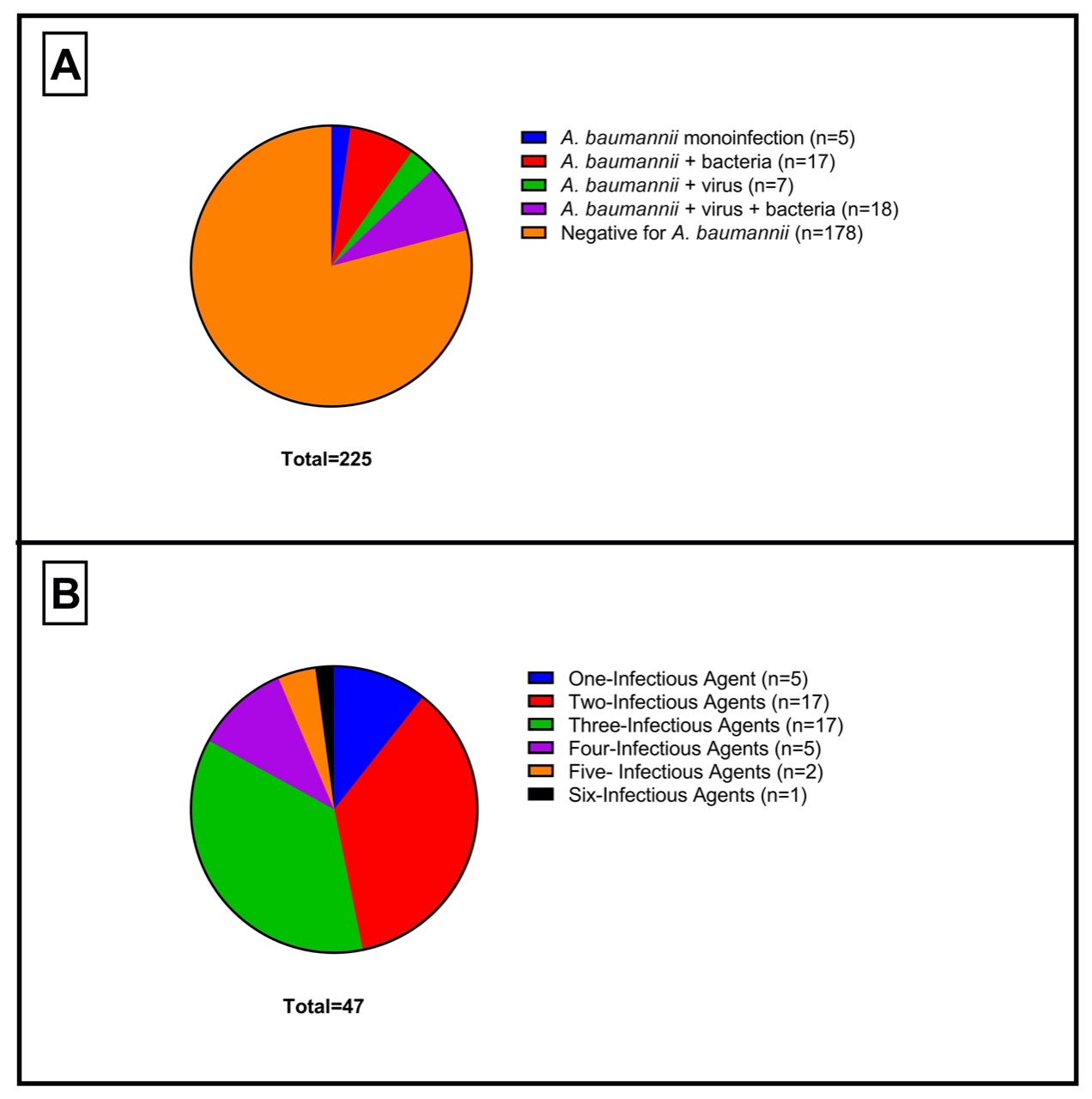

Supplement: Supplementary file 2 — Additional file 2: Fig. S1. A: Pattern of infection in the total population. B: Co-detection of pathogens in Acinetobacter baumannii positive children. [file 13104_2021_5826_MOESM2_ESM.jpeg]
